# PGV-1 Causes Disarrangement of Spindle Microtubule Organization Resulting in Aberrant Mitosis in HLF and HuH6 Cells Associated with Altered MYCN Status

**DOI:** 10.34172/apb.2024.058

**Published:** 2024-07-31

**Authors:** Nadzifa Nugraheni, Ummi Maryam Zulfin, Beni Lestari, Novia Permata Hapsari, Muthi Ikawati, Rohmad Yudi Utomo, Yusuke Suenaga, Yoshitaka Hippo, Edy Meiyanto

**Affiliations:** ^1^Cancer Chemoprevention Research Center, Faculty of Pharmacy, Universitas Gadjah Mada, Sleman, Yogyakarta, Indonesia.; ^2^Laboratory of Evolutionary Oncology, Chiba Cancer Center Research Institute, Chiba, Japan.; ^3^Laboratory of Macromolecular Engineering, Department of Pharmaceutical Chemistry, Faculty of Pharmacy, Universitas Gadjah Mada, Sleman, Yogyakarta, Indonesia.; ^4^Laboratory of Medicinal Chemistry, Department of Pharmaceutical Chemistry, Faculty of Pharmacy, Universitas Gadjah Mada, Sekip Utara, Sleman, Yogyakarta, Indonesia.; ^5^Department of Molecular Carcinogenesis, Chiba Cancer Center Research Institute, Chiba, Japan.

**Keywords:** PGV-1, HCC, MYCN, Microtubule disarrangement, Sorafenib

## Abstract

**Purpose:**

The HLF and HuH-6 cell lines represent hepatocellular carcinoma (HCC) with different characteristics in chromosome content that may give different drug responses. Here, PGV-1 was intended to challenge the growth-suppressing effect on HLF and HuH-6 and trace the molecular target mechanism of action compared to sorafenib.

**Methods:**

We applied MTT cytotoxic assay, colony forming assay, flow cytometry analysis, immunofluorescence assay and western blot assay.

**Results:**

PGV-1 exhibited cytotoxic effects on HLF and HuH-6 with IC-50 values of 1 µM and 2 µM, respectively, whereas sorafenib showed less cytotoxicity with IC-50 values of 5 µM and 8 µM respectively. PGV-1 suppressed the cell growth permanently but not for sorafenib. Sorafenib did not change the cell cycle profiles on both cells, but PGV-1 arrested the cells at G2/M with the characteristic of senescent cells and mitotic disarrangement. PGV-1 and sorafenib showed the same effect in downregulating p-EGFR, indicating that both compounds have the same target on EGFR activation or as Tyrosine kinase inhibitors. PGV-1 suppressed the MYCN expression in HuH-6 and HLF cells but stabilized cMYC-T58 indicating that even though the MYCN was downregulated, the cells maintained the active form of cMYC. In this regard, PGV-1 also stabilized the expression of PLK-1 and AurA.

**Conclusion:**

PGV-1 elicits stronger cytotoxic properties compared to sorafenib. The lower the MYCN expression, the higher the cytotoxic effect of PGV-1. PGV-1 abrogates cell cycle progression of both cells in mitosis through EGFR inhibition and stabilizes PLK-1 and AurA in correlation with the suppression of MYCN expression.

## Introduction

 Hepatocellular carcinoma (HCC) is usually found in the malignant stage and has deadly characteristics caused by its genomic instability and complex gene expression profiles leading to asymmetric and symmetric division.^1^ There are several karyotyping found in HCC, including hypotriploid and tetraploid, that could give different growth characteristics, drug response, and drug resistance.^2,3^ The hypotriploid cells tend to divide asymmetrically. In contrast, the hyper tetraploid could divide symmetrically, producing cancer stem cells that are less sensitive to dividing targeted anticancer agents.^4,5^ Therefore, this cancer type may relapse when the chemotherapeutics agent is withdrawn. Sorafenib is the standard chemotherapeutics agent for HCC but is categorized as the raising resistance drug.^3^ This phenomenon encourages the exploration of more anticancer agents that can provide a more effective effect with permanently cytotoxic activity.

 PGV-1 shows a permanently cytotoxic effect against K562 cells as well as MDA-MB-131 cells, which is subjected to correlate with its effect on ROS metabolizing enzymes leading to cell cycle arrest at mitosis, senescence, and apoptosis.^6,7^ This mode of action of PGV-1 makes it different from the other common cytotoxic agents that usually act on DNA dynamics in cell cycle progression. Therefore, PGV-1 is less toxic against normal cells and does not significantly affect animal models.^8^ PGV-1 also shows effectiveness in suppressing cell proliferation of hypotriploid cancer cells such as T47D and tetraploid cancer cells such as MCF7. This compound also gives a similar cytotoxic effect on several types of cancer cells, such as TNBC and non-TNBC,^7,9,10^ and in regular p53 and p53 mutant cancer cells.^11^ These broad-spectrum activities of PGV-1 on cancer types but on normal cells indicate that this compound has a unique mode of action on cancer cells that is interesting to further explore on some other specific types of cancer in HCC. So far, PGV-1 has been confirmed to disrupt the mitotic dynamics targeting prometaphase. The molecular signaling in the regulation of prometaphase is complex, involving MYCN, AurA, and PLK1, as well as microtubule arrangement. Therefore, PGV-1 may target some of the MYCN-related signals.

 This study explores the growth-suppressing potential of PGV-1 against HLF and HuH-6 cell lines. HLF represents a hypotriploid karyotype HCC, and HuH-6 represents a hypertetraploid HCC^12,13^ Both cells also show different MYCN expression statuses but have similar malignancy characteristics as HCC. HuH-6 exhibits a high moderate expression of MYCN, while HLF expresses a low level of MYCN.^14,15^ Despite the differences in MYCN expression, both HLF and HuH-6 cell lines perform symmetric or asymmetric division in 2D culture and highly express and activate EGFR to induce cell proliferation.^16^ Though sorafenib, a multi-kinase inhibitor, effectively inhibits both cell growth through disruption of MAPK signaling, which is also usually dependent on EGFR activation. However, in some cases, sorafenib could not eradicate the cancer cells due to the raising of resistance phenomena in cancer tissue. PGV-1, with its unique mechanism compared to sorafenib, could overcome this problem.

## Methods

###  Cell lines

 The liver cancer cells used in this study, HLF (JCRB0405) and HuH-6 (JCRB0401), were collected from Chiba Cancer Center Research Institute (CCCRI), Japan. Cells were grown in Dulbecco’s Modified Eagle Medium (DMEM, Gibco) culture medium with 1% penicillin-streptomycin (Gibco) and 10% fetal bovine serum (FBS, Gibco). Cells were cultured in a 5% CO_2_ incubator at 37 °C.

###  PGV-1 and sorafenib

 The Cancer Chemoprevention Research Center (CCRC), Faculty of Pharmacy, Universitas Gadjah Mada Indonesia, provided PGV-1 in 95% purity, while sorafenib was obtained from SIGMA (SML2653).

###  Cytotoxic assay

 The HLF and HUH-6 cells were seeded in 96-well-plates (6000 cells/well), incubated for 24 h, then treated for 24 hours and 48 hours with various concentrations of PGV-1 (up to 10 µM) and sorafenib (up to 50 µM) with appropriate culture conditions and medium (DMEM, 37 °C). The cytotoxic test was carried out with 10 µL CCK-8 (Dojindo, CK04) dissolved in a culture medium up to 100 µL (2-hour incubation), then analyzed at a wavelength of 450 nM.

###  Clonogenic assay

 Cells were grown in 6-well plates (15 000 cells/well) and incubated for 24 hours in complete medium and appropriate conditions. Expanded cells were then treated with PGV-1 (2 µM) and sorafenib (5 µM) for 48 hours of incubation. After that, the media containing the treatment compound was replaced with fresh new culture media, and incubation was continued for the next 14 days of culture. On day 14, cells were fixed with 4% paraformaldehyde and stained with a Gentian Violet solution (0.1%, Sigma). Analysis was done by calculating the number of surviving colonies using the Colony Area.

###  Cell cycle assay

 Cell cycle assays conformed to the manufacturer’s guidelines (Abcam 139418). Liver cancer cells were cultured at a density of 80% and treated with PGV-1 and sorafenib for 24 hours of incubation. Cells were harvested with TrypLE^TM^ (Gibco^TM^ Select Enzyme (1X), no phenol red) and washed twice with PBS 1x. The collected cell pellets were fixed with 66% ethanol (2 hours ), then passed with cold PBS 1x, and continued staining with a solution containing Propidium Iodide and RNAse at a concentration according to the protocol. Incubated in the dark for 30 minutes (37 °C), diluted with 400 µL PBS 1X, then analyzed using a FACSCalibur Flow Cytometer.

###  Immunostaining in mitosis assay

 Cells were first grown on glass coverslips and then treated for 24 hours with PGV-1. After treatment and washing with PBS 1x, cells were treated with 4% paraformaldehyde (PFA) in PBS to fix the cells. Then, a permeabilization solution was given with 0.2% Triton X-100 in PBS and blocked with 5% FBS in PBS. Cells were incubated with mouse anti-tubulin (T6199, Sigma-Aldrich) at 4°C (2–16 hours), followed by incubation with Alexa Fluor 488-conjugated anti-mouse IgG secondary antibody (Invitrogen) for 1 hour. Nuclear staining was achieved using DAPI (Nacalai Tesque) before mounting with Fluoroshield (ImmunoBioScience). Fluorescent cell images were obtained using a confocal microscope with a laser scanning system (LSM 880, Carl Zeiss, Oberkochen, Germany).

###  Senescence detection

 The 70% confluence of incubated cells on 24 well-plates for 24 h were subjected to treatment with PGV-1 incubated for 24 h. After discharging the media, the cells were washed with PBS 1x two times and then stained with X-gal as instructed by the manufacturer with slight modifications accordingly.^6^

###  Capillary western blotting (Abby)

 Analyses were conducted using the ProteinSimple Abby System. Whole lysate samples were thinned with 0.1 × Sample Buffer. Subsequently, 4 parts of the thinned sample were mixed with 1 part 5 × Fluorescent Master Mix, which includes 5 × sample buffer, 5 × fluorescent standard, and 200 mM DTT. The Fluorescent Master Mix incorporates three fluorescent proteins serving as a ‘ruler’ to standardize the distance for each capillary, as the molecular weight ladder is exclusively on the first capillary, and each vein operates independently. Following this denaturation step, the prepared samples, blocking reagent, primary antibodies (all antibodies diluted at 1:100 for Aurora A (Abcam 13824), PLK-1 (Abcam 17056). A biotinylated ladder was employed to provide molecular weight standards for each assay. Once the plate was loaded, the fully automated capillary system executed the separation electrophoresis and immunodetection steps.

## Results and Discussion

###  Cytotoxic properties of PGV-1 and sorafenib on HLF and HuH-6 cells

**Figure 1:**
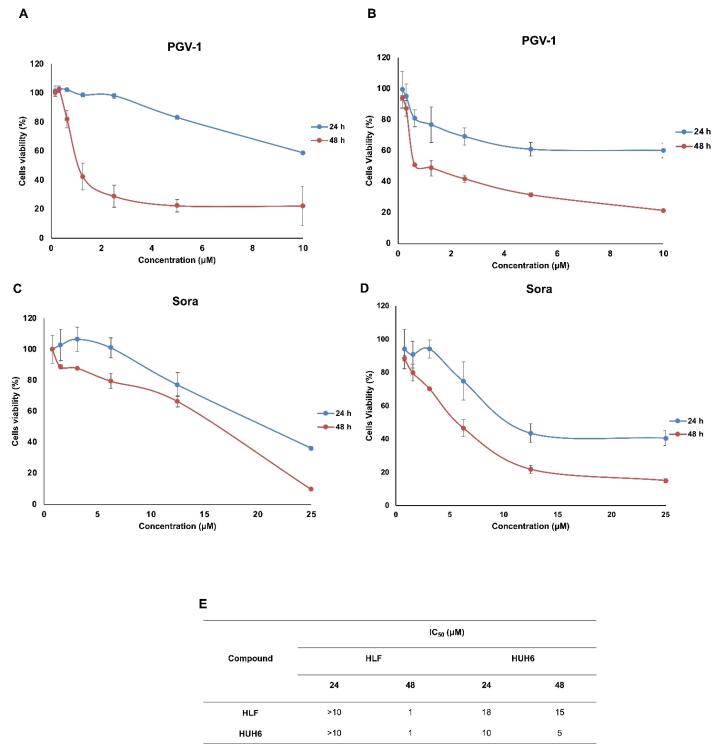
Cytotoxic profiles of PGV-1 (A&B) and sorafenib (C&D) on HLF and HuH6 cell lines. Cytotoxic assays were conducted for 24 and 48 h in serial concentration as indicated. The IC50 values (E) were presented based on the linearity profiles of the correlation between concentration and % cell viability with *P* value of < 0.05

 Exploration of the cytotoxic effects of PGV-1 and sorafenib on HCC cells revealed distinctive patterns of anti-proliferative activity. In a dose-response study, the IC_50_ of PGV-1 was lower than that of sorafenib in HLF and HUH-6 HCC cell lines following a 24 and 48-hour incubation period (Figure 1A-E). Notably, PGV-1 exhibited heightened cytotoxicity towards HLF cells, with low expression of MYCN^15^ and marked by a more significant reduction in cell viability than sorafenib.

**Figure 2:**
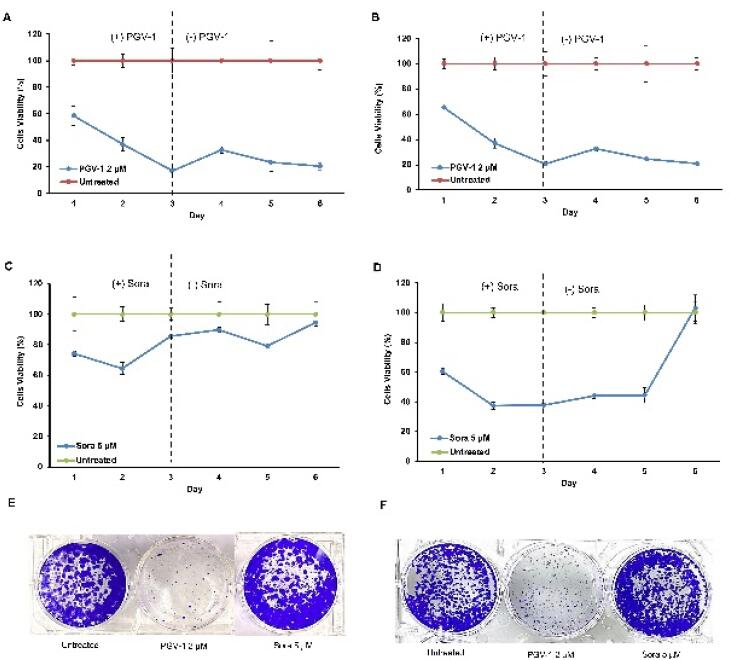
The persistence and Clonogenic assay of PGV-1 and sorafenib on HLF and HuH6 cell lines. Cells were grown and incubated with the drugs for 3 days then replaced the media without the drug to observe the viabilities up to 6 days (A: upper panel). Clonogenic assays were carried out as described in the method. The IC50 concentration of the compounds were applied and observed for 6 days of the cell viabilities (B: lower panel)

 Subsequent evaluation using a washout assay indicated that PGV-1 showed permanent arrest on both cells even though the compound was removed after 72 hours of incubation (Figure 2A, B). Besides, the clonogenic assay provided additional insights into the long-term effects of PGV-1 and sorafenib on colony-forming ability. PGV-1 significantly suppressed the clonogenic potential of both HLF and HUH-6 cells, highlighting its ability to inhibit cell proliferation irreversibly (Figure 2C, D). In contrast, sorafenib relapses the cell’s growth after withdrawing the drugs on day 3. Moreover, sorafenib exhibited less effect on clonogenicity, suggesting a reversible and milder inhibition of colony formation (Figure 2). These findings confirm the differences in the cytotoxic profiles of PGV-1 and sorafenib on HCC cells, with PGV-1 showing potent and irreversible anti-proliferative effects. At the same time, sorafenib exerts a comparatively milder and reversible impact on cell viability and colony-forming ability.

###  Cell cycle and mitotic profiles

**Figure 3:**
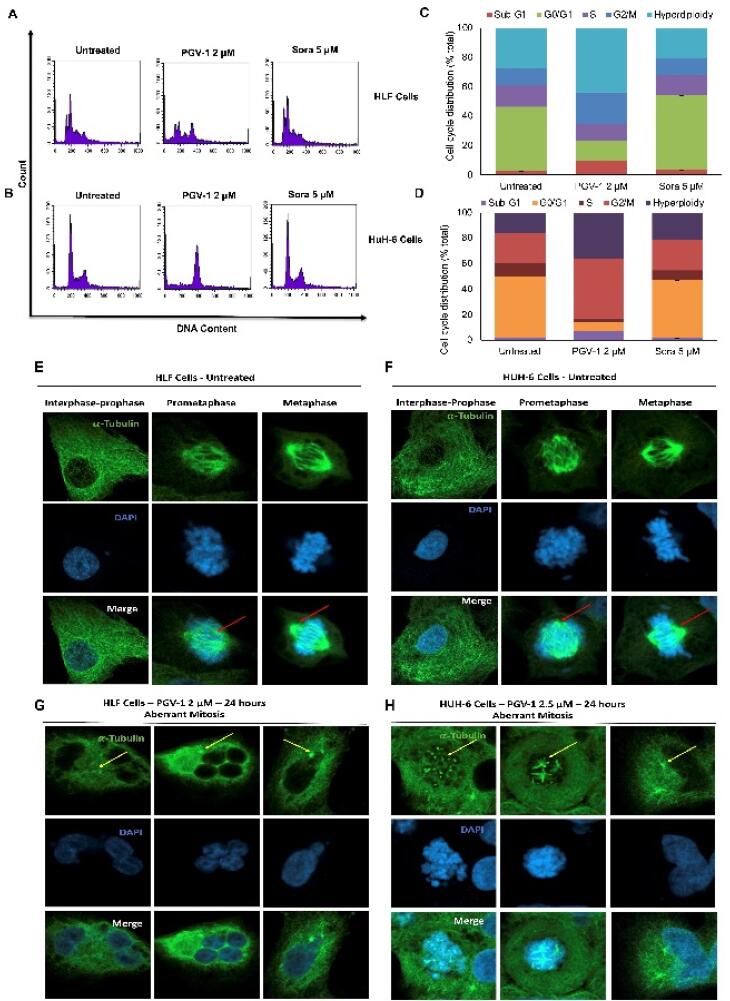
Cell cycle cell division profiles due to PGV-1 and sorafenib treatment on HLF and HuH6 cells. Cells in an appropriate amount were stained with Propidium Iodide (PI) and measured the DNA content using flow cytometry (A&B) and quantified of the 3 independent measurements (C&D). The mitotic appearance of the treated cells were observed using microscope fluorescence after being stained with an a-tubulin employment to detect the microtubule arrangement under confocal microscope (E,F,G&H). red arrows: arranged microtubule spindle; Yellow arrows: dis-arranged microtubule spindle

 Furthermore, we performed cell cycle analysis to elucidate the specific impact of PGV-1 and sorafenib on the cell cycle progression of HCC cells, providing further insights into the mechanisms underlying their different cytotoxic effects.As shown in Figure 3A-D, PGV-1 consistently triggers G2/M arrest. However, in HLF cells, an intriguing finding emerges with two splitting G1 phase peaks, implying chromosomal variations within a cell population. This occurrence may be attributed to the hypotriploid karyotype of HLF cells. Hyperpolyploid cell growth was also observed after treating PGV-1 in both cell lines. Nevertheless, sorafenib maintains a profile similar to that of untreated conditions. These findings imply that PGV-1 may solve the resistance of sorafenib in HCC cells, specifically in HLF and HUH-6, regarding cell cycle changes.

 More detailed observations on mitosis dynamics with tubulin immunofluorescence combined with DAPI staining revealed that the HLF and HuH-6 cells showed different appearances in the treatment of PGV-1. The untreated cells confirmed a normal cell division phenomenon with the controlled arrangement of microtubule spindle formation (Figure 3E-F). In contrast, the treatment of PGV-1 caused the disarrangement of microtubule spindle formation, causing mitotic abrogation in prometaphase. Although PGV-1 caused mitotic aberrant on both HCC cell lines, the arrested cells exhibited different characteristics. The HLF cells tend to collapse the cells into a giant polyploid, whereas the HuH-6 cells appeared in more tetraploid cell arrest conditions (Figure 3G-H). These results most likely align with the cell cycle data in which PGV-1 induced more polyploid in HLF but increased the G2/M phase in HuH-6. PGV-1 also causes mitotic catastrophe in several cell lines.^17,18^ The different effects of PGV-1 against HLF and HuH-6 cells may be correlated to their differences in chromosomal karyotypes.

###  Senescent cell and apoptosis profiles

**Figure 4:**
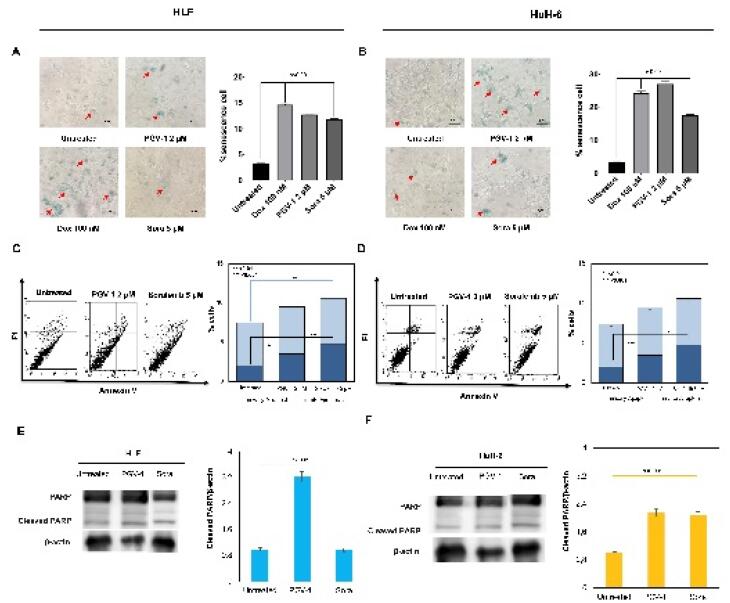
PGV-1 induced cell senescence and apoptosis of HLF and HuH6 cells. Cells were grown on 24 well plates and subjected to ꞵ-galactosidase detection as described in the method (A&B). Flow cytometry analysis was employed to detect apoptosis using Anexin V (C&D). Western blotting was implemented to detect the cleaved PARP as the markers of apoptosis evidence (E&F)

 We realized that PGV-1 selectively targets prometaphase, dis-arrangement of microtubules, and inducing mitotic arrest in HLF and HUH-6 cells. In some instances, mitotic arrest can serve as a trigger for inducing cells into a senescent state, leading to apoptosis. This evidence may happen when cells encounter difficulties completing mitotic division, which may activate salvage pathways or cell cycle arrest, leading to senescence. For this purpose, cells were treated with PGV-1 and sorafenib for 24 hours and tested for senescence-associated (SA)-β-galactosidase (gal) activity, a senescence marker. As shown in Figure 4D, PGV-1 induces SA-β-gal activity, which correlates with the quantification result of senescence evidence (Figure 4E). On the other hand, doxorubicin (as a positive control) and sorafenib also increase the senescence of both cells. Thus, PGV-1 at a lower concentration than sorafenib arrests cells at mitosis and induces senescence.

 Since the senescence cell is commonly connected to apoptosis, we then observed the apoptosis appearance of the cells after treatment with PGV-1 or sorafenib using annexin-V flow cytometry. PGV-1 and sorafenib slightly induced apoptosis in both cells. These results confirmed the cell cycle profiles, showing that the treatment of PGV-1 or sorafenib only increased cell accumulation in the sub-G1 phase to indicate cell death. However, we also identified that increased cleaved-PARP expression supports the apoptosis evidence. Therefore, PGV-1 or sorafenib treatment decreased the cell viability partly in correlation with apoptosis, and the suppression effect of PGV-1 was significantly supported by cell cycle arrest through mitotic dis-arrangement.

###  PGV-1 changes the protein expression of mitotic markers

**Figure 5:**
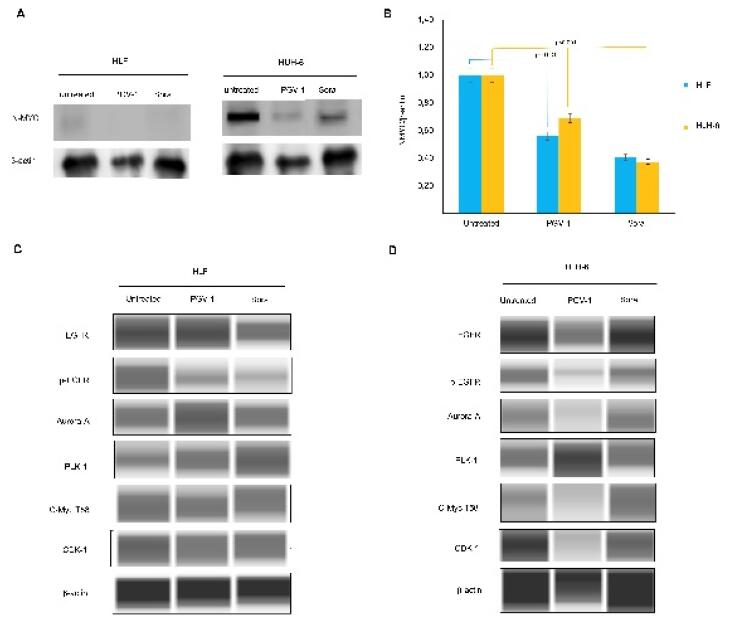
Protein expression due to PGV-1 and sorafenib treatment. Western blotting was conducted in two systems as described in the method; membrane transferred wet system (A&B) and capillary system (C&D) using the same samples which are the lysates from the treated cells with the respected sample

 To elucidate the molecular evidence underlying the physiological events caused by PGV-1, we analyzed the concerning protein expression. We found that PGV-1 significantly decreased MYCN protein expression of HLF and HuH-6 cells (Figure 5A-B). We noted here that even though the HLF cells were known to express MYCN, the N-MYC protein could rarely be detected at a low level. Furthermore, PGV-1 inhibited the phosphorylated EGFR expression comparable to sorafenib treatment, the well-known multi-kinase inhibitor. Since PGV-1 is well-known to affect mitosis progression, we also evaluated the central kinases involved in prometaphase and metaphase dynamics, namely PLK1, AurA, and CDK-1. Interestingly, PGV-1 increased the expression of PLK1 in both cells and AurA in HLF but decreased AurA and CDK1 levels in HuH-6. Meanwhile, PGV-1 slightly lowered the expression of c-MYC-T58 in both cell lines (Figure 5C-D).

 All these results demonstrate that PGV-1 as an anticancer provides consistent results. PGV-1 shows a robust cytotoxic effect on cancer cells, which is related to the cell cycle modulation in the mitotic phase. This observation also produces the same results, namely that PGV-1 exhibits strong growth suppression effects on HLF and HuH-6 cells and causes cell cycle arrest in mitosis. The new finding in this trial is that the cytotoxic effect of PGV-1 is stronger (more than 2 times) compared to the standard drug for liver cancer, sorafenib. Moreover, PGV-1 also shows a permanent growth suppression effect, while sorafenib still shows relapse after drug cessation. These results are interesting because PGV-1 can be expected to replace sorafenib for liver cancer (HCC), which is generally malignant. Therefore, we explore the essential differences between PGV-1 and sorafenib related to their physiological effects and molecular changes that occur due to the administration of these two compounds.

 In this study, we used 2 types of HCC cells, namely HLF and HuH-6, which differ in karyotype and MYCN expression.^13,15,19^ PGV-1 treatment on both types of cells generally gave the same pattern, namely PGV-1 caused cell cycle arrest in the G2/M phase. However, HLF cells show a different pattern to HuH-6. In each phase, 2 peaks appeared in HLF cells, while HuH-6 cells showed a regular peak pattern. This pattern with 2 peaks is a common characteristic of cells with a hypotriploid karyotype, such as T47D breast cancer cells,^20^ which is caused by some cells having a chromosome number of less than 2n. Interestingly, both cells accumulate in hyperpolyploid stages, which may be a marker effect of PGV-1 on cancer cells. In contrast, sorafenib did not change the cell cycle profile at all cell types up to 48 hours of treatment, indicating that sorafenib did not affect cell cycle regulation. Sorafenib is a multi-kinase inhibitor and can lead to apoptosis in cancer cells.3 On the other hand, PGV-1 consistently affects the mitotic phase of the cell cycle, although its specific molecular target is currently unknown. Our experiments confirmed that PGV-1 induced accumulation of the cells in prometaphase and showed disarrangement of microtubule spindle formation. Even though the effects of PGV-1 on HLF and HuH-6 give different phenomena in cell morphology, both cell types are arrested in the same mitosis phase at prometaphase, leading to mitosis abrogation, polyploid, and apoptosis. Nevertheless, PGV-1 has shown better efficacy than sorafenib, likely due to differences in molecular targets in cell division. In this regard, the disarrangement of microtubule spindle formation should be noted as a signature of the PGV-1 effect on cancer cells, making this compound exert a better anticancer property than sorafenib.

 Sorafenib still had a good cytotoxic effect on HLF and HuH-6 cells even though its potency was lower than PGV-1. Unfortunately, sorafenib also shows a relapse phenomenon, the new growth of cancer cells after drug cessation, even after removing the drug. This effect is different from PGV which shows sustained suppression of cancer cell growth. This phenomenon is another advantage of PGV-1 over sorafenib, also demonstrated in K562 cells compared to Gleevec, the standard chronic myeloid leukemia (CML) drug.^21^ PGV-1 shows a permanent growth-suppressing effect due to the irreversible interaction of PGV-1 with essential proteins, such as ROS metabolizing enzymes, in cancer cells.^7^ However, whether the interaction of sorafenib with protein kinases is also reversible still requires further study. Another possibility is that changes in the expression of survival genes in cancer cells, such as stemness characteristics, cause the emergence of resistant cells. Hence, the cancer cells become no longer sensitive to sorafenib. These results indicate that PGV-1 provides an alternative way to overcome the problem of cancer cell resistance to chemotherapy agents, especially in HCC, and thus requires further exploration.

 We also noted that PGV-1 and sorafenib exerted similar effects on inhibiting EGFR phosphorylation, indicating that PGV-1 also has tyrosine kinase inhibitory activity. These results prove the previous hypothesis that molecularly docking PGV-1 can interact with the kinase domain in HER2 and EGFR.^8^ This finding is important because it can provide a reason for the cytotoxic activity of PGV-1 in cancer cells because, in general, cancer cells undergo increased activation of the EGFR pathway.^22^ Increased expression and activation of EGFR was also found in HCC, including HLF and HuH-6 cells.^23^ Meanwhile, PGV-1 also showed activity to decrease MYCN expression in HLF cells but increased or stabilized PLK1 and AurA expression. PLK1 and AurA proteins play an important role in mitotic dynamics from prophase to metaphase.^24^ High expression of PLK1 and AurA seems to be a sign that the cell is still active in prometaphase or metaphase, and this could occur possibly because these two proteins are stabilizing or not being degraded. This phenomenon suggests that PGV-1 might inhibit the PLK1 and AurA degradation pathways, so cells stop in prometaphase. Another fact that supports this incident is the high expression of the cMYC-T58 protein, which indicates that this protein is not degraded and continues to work in prometaphase and metaphase. However, further study is still required on how this condition relates to EGFR downregulation. In addition, N-MYC and c-MYC are involved in stemness formation that led to drug resistance.^25^ but this condition did not affect the growth suppression effect of PGV-1.

 These observations provide a clearer picture of PGV-1’s anticancer activity, which is better than sorafenib’s on HLF and HuH-6 cells. PGV-1 provides good prospects as a suppressor of HCC growth regardless of the karyotype because it can inhibit EGFR and mitotic dynamics at metaphase, differentiating it from other anticancer agents. The specific mechanism of PGV-1 in overcoming HCC resistance, which is generally related to stemness characteristics, could be an interesting research focus in the future. All the results of these observations provide a clearer picture of the anticancer activity of PGV-1 which is better than sorafenib on HLF and HuH-6 cells. PGV-1 provides good prospects as a suppressor of HCC growth regardless of the karyotype because PGV-1 can not only inhibit EGFR but also inhibit mitotic dynamics at metaphase, which differentiates it from other anticancer agents. The specific mechanism of PGV-1 in overcoming HCC resistance which is generally related to stemness characteristics could be an interesting research focus in the future.

## Conclusion

 PGV-1 inhibits HLF and HuH-6 cell growth better than sorafenib with permanently inhibitory characteristics. HLF, the hypotriploid HCC, shows more sensitivity to PGV-1 and sorafenib compared to HuH-6 cells, but both cells exhibit the same cell cycle arrest profiles, that are G2/M arrest, especially in prometaphase, against PGV-1 but not to sorafenib. The mitotic arrest of the cells by PGV-1 treatment is probably due to the disarrangement of microtubule spindle formation. It possibly correlates to the effect of PGV-1 on the downregulating of EGFR and MYCN and the upregulating of PLK1 and AurA. PGV-1 may have better prospects than sorafenib as an anticancer agent for HCC.

## Acknowledgments

 We thank “Beasiswa Pendidikan Indonesia (BPI)’’ granted to Nadzifa Nugraheni for the partly supported funding in student exchange programs between Universitas Gadjah Mada and Chiba Cancer Center Research Institute (CCCRI). We are also thankful to CCCRI for the Laboratory facility and supervision in the student internship program.

## Competing Interests

 All authors declare that they have no competing interests.

## Ethical Approval

 Not applicable.
